# Properties of residual titanium dioxide nanoparticles after extended periods of mixing and settling in synthetic and natural waters

**DOI:** 10.1038/s41598-017-09699-9

**Published:** 2017-08-30

**Authors:** Chunpeng Zhang, Jenyuk Lohwacharin, Satoshi Takizawa

**Affiliations:** 1 Jilin University, Key Laboratory of Groundwater Resources and Environment, Ministry of Education, No.2519, Jiefang Road, Changchun, 130021 China; 2 Chulalongkorn University, Department of Environmental Engineering, Faculty of Engineering, Phayathai Rd., Wangmai Pratumwan, Bangkok, 10330 Thailand; 3 The University of Tokyo, Department of Urban Engineering, Graduate School of Engineering, 7-3-1 Hongo, Bunkyo-ku, Tokyo, 113-8656 Japan

## Abstract

Titanium dioxide nanoparticle (TiO_2_ NP) discharged into water bodies can affect ecosystems and human health adversely. We studied the properties of residual TiO_2_ NPs with and without gentle mixing (to simulate a natural environment more closely) and after settling for 12-h periods. Surface complexation, dynamic particle size changes, and TiO_2_ NP destabilization in synthetic and lake waters were investigated. The accumulation of inert ions (Na^+^ and Cl^−^) in the diffuse layer which was not discussed in other studies was supposed to be the main reason that aggregation occurred slowly and continuously. PO_4_^3−^ stabilized and destabilized TiO_2_ NPs at 10 mM and 100 mM, respectively. Destabilization occurred because high ionic strength overwhelmed increased negative charges of TiO_2_ NPs by complexation with PO_4_^3−^. TiO_2_ NP destabilization was achieved in approximately 12 h in synthetic and lake waters, and is attributed to the slow diffusion of ions into aggregates. Despite the presence of moderately high concentrations of natural organic matter, which tends to stabilize TiO_2_ NPs, the addition of 20 mM PO_4_^3−^ destabilized the TiO_2_ NPs in lake water. Smaller aggregate sizes formed compared with those before destabilization, which indicates that stable residual TiO_2_ NPs could exist in aquatic environments after extended periods.

## Introduction

The increased production and widespread use of nanoparticles has raised concerns regarding their environmental fate and ecological toxicity^1, 2^. Of the various nanoparticles (NPs) that are manufactured industrially, titanium dioxide nanoparticles (TiO_2_ NPs) are produced in the highest amounts for various uses, such as in cosmetics, in personal-care products, as food additives, in paints, and in coatings^3–6^. Most TiO_2_ NPs are discharged into sewerage systems, and 10–100 μg/L of TiO_2_ remains in secondary effluents^1, 7, 8^.

The stability of NPs in various water matrices determines their aggregate size, which affects their fate and toxicity^9, 10^. Hence, it is imperative to investigate NP destabilization in natural water environments^1, 11–13^. Factors that affect NP stability include their physico-chemical characteristics, such as surface chemistry^14^, particle size^15^, structure, and composition^16^. Their stability is also affected by water properties, such as the solution pH, ionic strength (IS) and ionic species, and dissolved organic matter (DOM)^11, 13, 17–20^.

Cations such as Na^+^ and Ca^2+^ have been reported to destabilize TiO_2_ NPs in synthetic waters, i.e., pure water that contains electrolytes and organic matter, by compaction of the electric double layer or by complexation and surface-charge destabilization^13, 17, 19, 21–23^. Thus far, the detailed mechanism of complexation of these ions has not been explained. The effects of organic matter, e.g., surfactants^24^, alginate^17^, Suwannee River natural organic matter^23^, and Suwannee River fulvic acid^25^, combined with inorganic ions such as Na^+^ and Ca^2+^ have been investigated. Zhang *et al*. reported that 60 mM Ca^2+^ destabilizes natural organic matter-coated metal-oxide NPs^23^. Ca^2+^ exhibits a more pronounced effect toward hematite destabilization, but Na^+^ addition affects destabilization by Ca^2+^ adversely, which is attributed to competition for adsorption sites^17^. TiO_2_ NP destabilization by Suwannee River fulvic acid has been reported to be enhanced by PO_4_^3−^ addition^18^, while the surface charge of TiO_2_ NPs is retained. These results cannot be explained by conventional (or classic) models. Keller *et al*. measured electrophoretic mobility as an indicator of NP destabilization^20^, but properties of the suspended NPs after aggregation and settling were not reported.

Although larger NP aggregates can settle and be removed from bulk solutions, residual NPs in supernatants most likely affect ecosystems and human health by entering drinking water sources^9^. Hence, it is imperative to investigate the properties of NPs that remain suspended after destabilization and settling in natural water bodies. Previous studies have assumed that surface complexation, or adsorption, of ions and organic matter is spontaneous, where complexation and equilibration are rapid^26^. Thus, NP samples have been analyzed less than 0.5 h after vortex mixing and/or sonication^9, 17, 19, 22, 27^ despite strong mixing conditions being unlikely in natural water bodies. Some studies have reported that NP aggregation and settling is slow, and can take up to 24 h^10, 14, 20, 25^; inorganic ion adsorption destabilizes hydrous oxides with a range of equilibrium times from minutes to weeks^28^. Furthermore, it is still unclear how properties such as particle size and the zeta potential of TiO_2_ NPs change dynamically in the water body.

We investigated the effect of extended mixing periods on surface complexation, dynamic particle size change, and TiO_2_ NP destabilization in synthetic and natural waters. We collected TiO_2_ NPs from supernatants that were generated from settled samples that had been subjected to no mixing and to gentle mixing. Studies without mixing represent quiescent water bodies, whereas those with gentle mixing represent mildly mixed water bodies. The total settling time was 12 h, with an initial static period of 0.5 h being followed by a gentle mixing period of 11.5 h. The experimental results of the destabilization of TiO_2_ NPs in synthetic waters were used to discuss the destabilization of TiO_2_ NPs during extended mixing in natural lake waters.

## Materials and Methods

### Preparation of TiO_2_ NP Stock Solution

TiO_2_ NPs were of the anatase form with a primary diameter of approximately 25 nm (Sigma Aldrich, Saint Louis, MO, USA) and a Brunauer–Emmett–Teller specific surface area of 48.7 m^2^/g as measured by N_2_ adsorption (BELSORP Mini-II, MictroacBEL Corp., Japan). As-received NPs were aggregates of slightly irregularly shaped spheres (see transmission electron micrographs, Figure S1, Supplementary Information)^14^. In this study, X-ray diffraction (XRD) analysis was performed using a Rigaku MiniFlex II Desktop X-Ray diffractometer. Diffraction angle was scanned in the range of 10–80°. Scanning speed was 2°/min. and sampling width was 0.02°. Phase identification and percent (%) crystallinity were determined with the XRD pattern processing, identification and quantification software (MDI Jade 7 v.7.5.11, Materials Data, Inc., Japan), which has been installed in the diffractometer. The data base is referred to International Centre for Diffraction Data (ICDD). XRD results indicated that the anatase crystallinity was greater than 99% (Figure S2). A TiO_2_ NP stock solution that contains a stable TiO_2_ NP suspension was prepared by dispersing 25 mg of TiO_2_ NPs in 50 mL of Milli-Q water, which was sonicated at 150 W for 30 min to disperse large agglomerates^24^. The solution was placed in the dark for 1 week to remove the settleable fraction. The concentration of TiO_2_ NPs in the supernatant was quantified as described in the analytical method section. The supernatant with stable TiO_2_ NPs was transferred to a new vial, and the concentration was adjusted to 100 mg/L using Milli-Q water (hereafter, termed TiO_2_ NP stock solution). In these experiments, TiO_2_ stock solution (12.5 mL) was diluted by either Milli-Q water or lake water (32.5 mL), and then pH was adjusted by HCl or NaOH (5 mL).

### Stability of TiO_2_ NPs in Static Synthetic Water

Milli-Q water that contains 25 mg/L of TiO_2_ NPs was adjusted to pH 9 and an IS of 0.1 mM or 15 mM by NaCl addition. pH adjustment to alkaline condition (pH ~ 9) is to represent natural eutrophic lake water. This solution (400 mL) was added into a beaker and kept static, i.e., with no mixing, at 25 ± 1 °C for 12 h. Water samples (5 mL) were removed from the top layer of the solution after every 30 min, and the particle-size distribution was analyzed by dynamic light scattering (DLS) as described below. Changes in the particle-size distribution during destabilization under static conditions for 12 h were represented by using contour maps of the particle-size distribution as drawn with SigmaPlot 12.5 (Systat Software, Inc.) and a method reported previously^19^.

### Complexation of Na^+^ and Cl^−^ at Various pH Levels

The TiO_2_ NP stock solution was diluted using Milli-Q water to obtain a solution that contains 25 mg/L TiO_2_ NP, and which was comparable with that reported previously^20, 24, 29^. The pH was adjusted using HCl or NaOH to the pH indicated in Table 1. No additional electrolyte was added to adjust the IS. After the pH had been adjusted, samples were kept static for 0.5 h, and then 5 mL of the sample was collected for pH, particle size (nanoparticle tracking analysis (NTA) method), and zeta potential analysis. Immediately after the initial sampling, solutions were placed on a rotary shaker and were mixed gently at 120 rpm for 11.5 h. After 10 min of settling (i.e., at 40 min after the start of the test and 12 h 10 min after the start of the test), 5 mL of supernatant was removed to analyze the aforementioned parameters. All experiments were conducted at 25 ± 1 °C in a temperature-controlled incubator. The amount of Na^+^ and Cl^−^ that complexed with TiO_2_ NPs was quantified from the difference in concentrations of these ions at 0.5 h and 12 h. These experiments were conducted in duplicate. Error bars on the figures represent standard deviations (n = 3).

**Table 1:** Experimental conditions.

Exp. no.	Factors examined	Water sample, ionic strength, pH adjustment (buffer)	Initial pH	Solution volume (mL)
1	Particle-size distribution change over 12 h	Milli-Q water; NaCl 0.1 mM or 15 mM; pH adjusted by NaOH.	9.0	400
2	Effect of cation species and ionic strength on stability	NaCl or CaCl_2_; IS 0.3 mM or 15 mM	5.4–6.3	50
3	Phosphate concentration	K_2_HPO_4_; 0.1 mM,1 mM, 10 mM, 100 mM; no pH adjustment	8.4–9.2	50
4	Effect of Na^+^ and Cl^−^ complexation on solution pH and zeta potential of TiO_2_ NPs	Milli-Q water; no IS adjustment; pH adjustment by HCl or NaOH	3.9, 5.0, 6.9, 8.9, 10.1	50
5	pH and buffer solutions in lake water	CH_3_COOH/CH_3_COONa	4.5	50
		Na_2_HPO_4_/NaH_2_PO_4_	6.5	
		Milli-Q (no buffer)	6.8	
		Control (no buffer)	7.3	
		H_3_BO_3_/Na_2_B_4_O_7_·10H_2_O	9.0	
		*Buffer concentration: 20 mM		

Note: Initial concentration of TiO_2_ NPs was 25 mg/L in all experiments. The temperature was maintained at 25 ± 1 °C in an incubator.

### Effects of Ionic Species and Ionic Strength on TiO_2_ Stability

We selected NaCl and CaCl_2_ as these can be commonly found in natural water. The effects of ionic species and IS were evaluated using NaCl and CaCl_2_ at NaCl concentrations of 0.3 mM or 15 mM and CaCl_2_ concentrations of 0.1 mM or 5.0 mM at pH 5.4–6.3. The TiO_2_ NP concentration was adjusted to 25 mg/L as in the previous experiments, and destabilization experiments were conducted using the same method as in previous experiments with a variation of pH; namely, after being kept static for 0.5 h, solutions were mixed gently at 120 rpm for 11.5 h at 25 ± 1 °C. Supernatant samples were removed after 10 min of settling at 0.5 h and at 12 h for the analyses.

We also used PO_4_^3−^ as it is contained in detergents and thus in effluents from wastewater treatment plants. In experiments on the effect of PO_4_^3−^ concentration, the PO_4_^3−^ concentration was varied from 0.1–100 mM in TiO_2_ NP-containing Milli-Q water. The solution pH was 8.4–9.2, without any adjustment in pH to prevent changes to the IS. After the PO_4_^3−^ concentration had been adjusted, destabilization experiments were conducted by the same method as described previously. These experiments were conducted in triplicate. Error bars on the figures represent standard deviations (n = 3).

### Stability of TiO_2_ NPs in Lake Water

A water sample from Lake Kasumigaura, Ibaragi, Japan, was filtered using a 0.45-μm polytetrafluoroethylene (PTFE) membrane. The filtrate contained 3.5 mg/L of dissolved organic carbon (DOC) with an IS of 2.66 mM (detailed information in Table S1). This sample was placed in a refrigerator at 4 °C until use. After dispersing TiO_2_ NPs at 25 mg/L, the lake water pH was adjusted to between 4.5 and 9.0 using 20 mM buffer solutions, as shown in Table 1. The lake water sample was kept static for 0.5 h, followed by gentle mixing on a rotary shaker at 120 rpm at 25 ± 1 °C to allow for nanoparticle and ion or organic matter contact and complete diffusion in the sample. Samples (5 mL) were collected from the lake water at 0.5 h, 1.0 h, 3.0 h, 6.0 h, and 12.0 h to determine the concentrations of TiO_2_ NPs, particle size, and zeta potentials.

### Analytical Methods

Dynamic light scattering (DLS, Nanotrac^TM^ 150, Nikkiso, Japan) and NTA (Nanosight LM10 system, Nanosight, UK) were used to monitor changes in the particle-size distribution of the TiO_2_ NPs in synthetic or natural solutions. NTA can measure refractive particles (e.g., ceramics and metal oxides, including TiO_2_ NPs) by tracking individual spots of laser-light reflection by particles that are suspended in solution. The hydrodynamic diameter was estimated by temporal evolution of the scattering light intensity and using the Stokes–Einstein equation. The output signal was analyzed mathematically by using Microtrac® Windows software to provide a volume- and number-based particle-size distribution using the Lorenz–Mie theory. The zeta potential of the NPs was measured using an ELS SF-8000 system (Otsuka Electronics, Japan). The TiO_2_ NP concentration was quantified using a spectrophotometer (U-2010, Hitachi Ltd., Japan) at 660 nm^33^. The Na^+^ concentration was quantified by atomic absorption spectrophotometer (AA6200, Shimadzu, Japan), and the Cl^−^ concentration was measured by ion chromatography (861 Advanced Compact IC, Metrohm AG, Switzerland). Fluorescence excitation–emission matrix profiles were measured on a fluorescence spectrophotometer (F-4500, Hitachi, Japan) at excitation (Ex) and emission (Em) wavelength ranges of 220–450 nm and 230–550 nm, respectively, at an interval of 5 nm.

### DLVO Interaction Energy

The DLVO interaction energy of TiO_2_ NPs in each solution was calculated by using the following equation and assuming identically sized spheres^14, 21^:

1 $$\begin{array}{rcl}{\Phi }_{net} & = & \frac{64\pi {R}_{s}{n}_{\infty }{k}_{{\boldsymbol{B}}}T}{{\kappa }^{2}}\exp (-\kappa d)\times (\frac{\exp (\frac{ze\psi }{2{k}_{{\boldsymbol{B}}}T})-1}{\exp (\frac{ze\psi }{2{k}_{{\boldsymbol{B}}}T})+1})\\  &  & -\frac{{\rm{A}}}{6}[\frac{2{{R}_{s}}^{2}}{{d}^{2}+4{R}_{s}d}+\frac{2{{R}_{s}}^{2}}{{d}^{2+}+4{R}_{s}d+4{{R}_{s}}^{2}}+\,\mathrm{ln}(\frac{{d}^{2}+4{R}_{s}d}{{d}^{2+}+4{R}_{s}d+4{{R}_{s}}^{2}})]\end{array}$$

where *R*_*S*_ is the particle radius (m) estimated from the number-based mean value by NTA, *n*_*∞*_ is the bulk electrolyte number concentration (m^−3^), *k*_*B*_ is the Boltzmann constant (1.38 × 10^−23^ J K^−1^), *T* is the absolute temperature (298.15 K), *κ* is the inverse of the Debye length (m^−1^) as calculated by the dissolved ion concentrations, *d* is the separation distance between NPs, *z* is the valence of ions, *e* is the elementary charge (1.60 × 10^−19^ C), *ψ* is the zeta potential (V) measured during each experiment, and *A* is the Hamaker constant for anatase TiO_2_ (3.40 × 10^−20^ J) (Table S2 **)**.

## Results and Discussion

### Particle Size Changes

**Figure 1:**
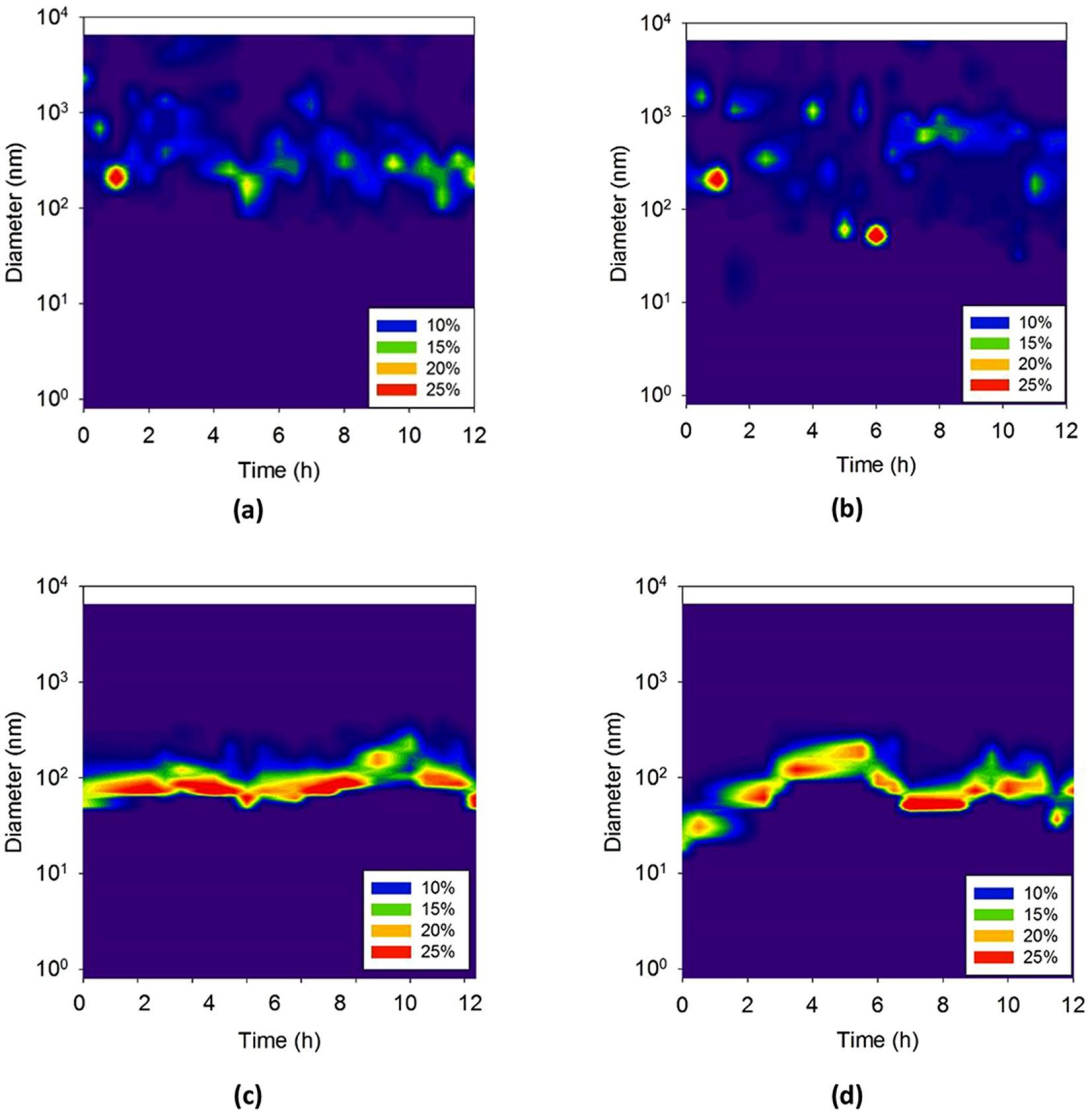
Changes in particle sizes during destabilization for 12 h. Volume-based particle counting (**a**) 0.1 mM, (**b**) 15 mM; number-based particle counting (**c**) 0.1 mM, (**d**) 15 mM, pH 9. The scale refers to the proportion of particles of a particular size in the solution.

Figure 1 shows the number- and volume-based particle-size distributions during destabilization for 12 h. The volume-based counting of particles by DLS exhibits a polydisperse distribution with spots of different particle sizes^19^. The variation in size was greater in 15 mM NaCl, which indicates that a greater destabilization occurred than in 0.1 mM NaCl. Although no clear difference was visible in terms of the spot size or location for the 0.1 mM NaCl and 15 mM NaCl solutions, a slightly larger number of spots was observed near or above 1000 nm in the 15 mM NaCl solution compared with those in the 0.1 mM NaCl solution. This result is attributed to the fact that, when large aggregates form, they appear as spots in volume-based counting, whereas, small particles go undetected. Therefore, in volume-based particle counting, the particle-size distribution depends on the detection of larger particles, which exist in smaller numbers, compared with smaller particles. Hence, the emergence of large particle spots and high levels of polydispersity (as was visible in the 15 mM NaCl solution) in the volume-based particle counting indicate a high extent of particle destabilization.

In contrast with the volume-based particle counting, number-based particle counting by DLS exhibited a band near 100 nm in the 0.1 mM NaCl solution for 12 h, which is attributed to the emergence of a small number of large particles that are relatively insignificant in number. The band level showed that the particle size did not change in the 0.1 mM NaCl solution, although a small increase was observed at 9–10 h. An increasing trend in particle-size distribution occurred in the 15 mM NaCl until almost 6 h, followed by a decrease to ~80 nm. The sudden disappearance of the increasing band is attributed to particle aggregation, settling, and the removal of particles, which results in fewer remaining particles. After 12 h, a smaller particle size results after destabilization, suggesting that the aggregates are smaller after destabilization and settling.

### Variation in pH

**Figure 2:**
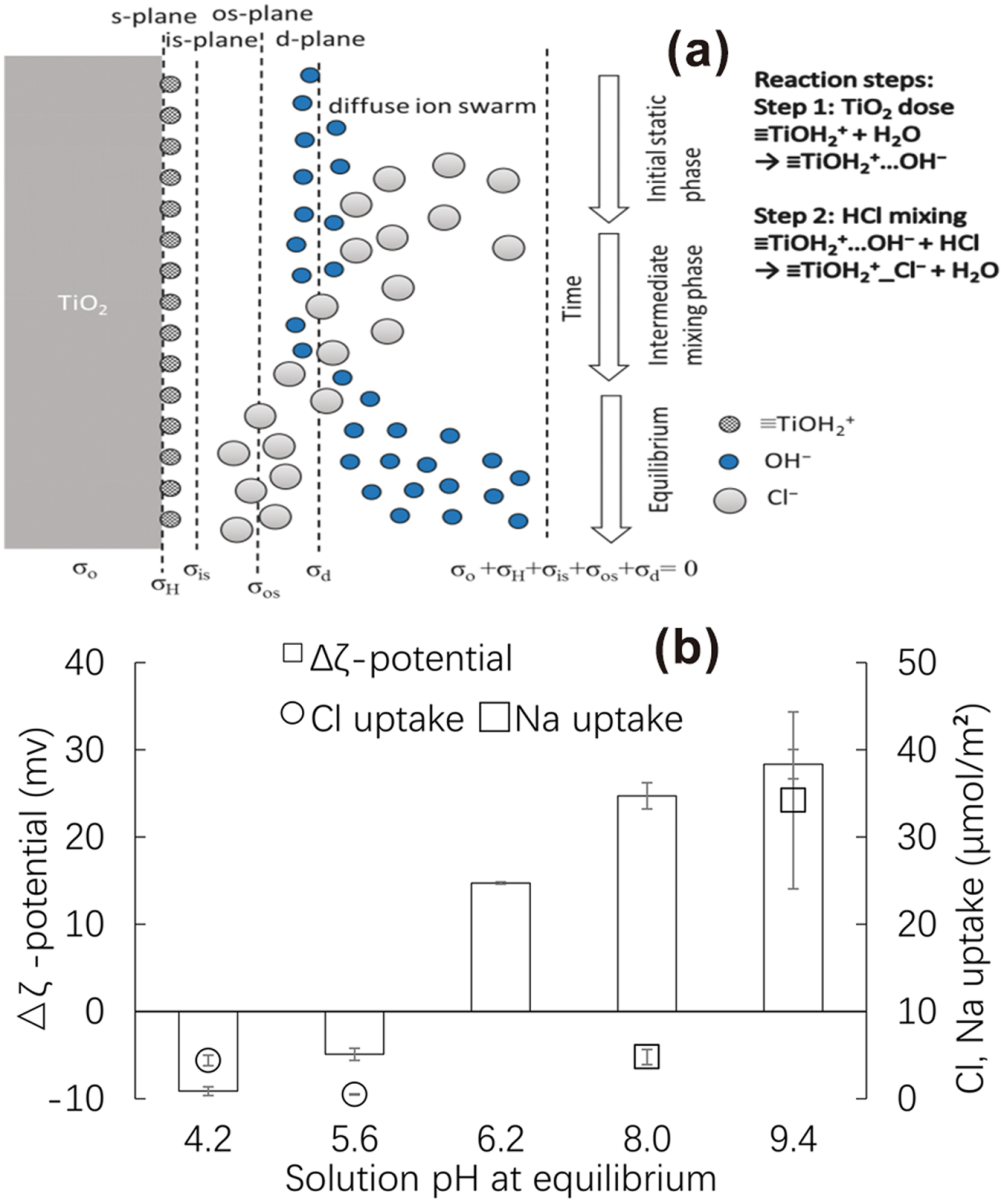
(**a**) Schematic diagram of chloride complexation at pH less than pH_pzc_. (**b**) Chloride or sodium uptake and changes in zeta potential after 12 h of gentle mixing.

Figure 2(b) shows the amounts of Na^+^ and Cl^−^ adsorbed on the TiO_2_ NPs and the changes in zeta potential at various pH values. At a high pH, large amounts of Na^+^ were adsorbed because of the high Na^+^ solution concentration from NaOH dissociation. Similarly, at a low pH, large amounts of adsorbed Cl^−^ were observed because of the high HCl dose. Janusz measured the adsorption of Na^+^ and Cl^−^ on rutile TiO_2_ using radio isotopes and observed the same pH dependence and Na^+^ and Cl^−^ complexation with rutile TiO_2_, for the same Na^+^ and Cl^−^ concentrations^30^. Thus, the pH dependence of Na^+^ and Cl^−^ adsorption is attributed to surface complexation reactions with alkaline p*K*_Na_ and acidic p*K*_Cl_ values. Despite the same trends in pH dependence, the amounts of Na^+^ and Cl^−^ complexed with anatase TiO_2_ NPs in this study were significantly greater than those complexed with rutile TiO_2_^30^, which may be attributed to differences in crystal forms, particle sizes, and experimental conditions. The small size of the primary particles in our study, i.e., ~25 nm, may affect the amount of Na^+^ and Cl^−^ that is complexed, as it has been suggested that the properties of NPs smaller than 30 nm differ significantly from those of bulk materials^31^.

The accumulation of inert ions (Na^+^ and Cl^−^) in the diffuse layer which was not discussed in other studies was supposed to be the main reason that aggregation occurred slowly and continuously. Ion complexation is considered to be spontaneous and rapid^26^; thus, in several studies, the NP surface charge was determined after mixing^17, 19, 27^. However, herein, we observed that complexation and equilibration between TiO_2_ NPs and the solution was incomplete after 0.5 h under static conditions, during which molecular diffusion predominated. After gentle mixing for 12 h, the TiO_2_ NPs and the solution reached equilibria, at which time, Na^+^ underwent complexation at a pH above the point of zero charge (pH_pzc_), whereas Cl^−^ underwent complexation at a pH below the pH_pzc_. Such slow destabilization and settling processes have been reported previously^10, 14, 20, 25^, although the amounts of ions complexed with the NPs have not been measured. Zhang *et al*. conducted aggregation and settling experiments using various metal-oxide NPs in a 100 mM MgCl_2_ solution^14^, and concluded that a long mixing time of 8–24 h is required to enhance the settling efficiency of NPs. Keller *et al*. reported that NP aggregation and sedimentation are slow^20^, and require more than several hours. The reason for this difference between rapid and slow aggregation in previous studies is unclear, and may result from the different experimental conditions used. Zhang *et al*. attributed the slow aggregation and settling to mechanisms of orthokinetic flocculation^14^. Baalousha estimated that diffusion-limited adsorption of Suwannee River humic acid into fractal aggregates of iron NPs resulted in the slow adsorption of Suwannee River humic acid^10^. Based on these references, the slow complexation of Na^+^ and Cl^−^ on the surface of TiO_2_ NPs in our study is assumed to result from: i) the static and gentle mixing conditions, and ii) diffusion-controlled complexation into TiO_2_ NP aggregates.

When Na^+^ and Cl^−^ adsorb on metal oxides, Na^+^ and Cl^−^ may form outer-sphere complexes with these oxides^26, 30^. Hence, the zeta potential shifts toward high positive values when Na^+^ is complexed at a pH higher than the pH_pzc_, whereas it shifts toward negative values when Cl^−^ is complexed at a pH lower than the pH_pzc_. The changes in zeta potential were greater for Na^+^ adsorption compared with those for Cl^−^ adsorption, which is attributed to the fact that the amount of adsorbed Na^+^ is greater than that of adsorbed Cl^−^. The surface complexation of Na^+^ and Cl^−^ is given as:

$${\rm{at}}\,{\rm{pH}} > {{\rm{pH}}}_{{\rm{pzc}}}\equiv {{\rm{TiO}}}^{-}\ldots {{\rm{H}}}^{+}+{{\rm{Na}}}^{+}=\equiv {{\rm{TiO}}}^{-}\_{{\rm{Na}}}^{+}+{{\rm{H}}}^{+}$$

$${\rm{a}}{\rm{t}}\,{\rm{p}}{\rm{H}} < {{\rm{p}}{\rm{H}}}_{{\rm{p}}{\rm{z}}{\rm{c}}}\equiv {{{\rm{T}}{\rm{i}}{\rm{O}}{\rm{H}}}_{2}}^{+}\ldots {{\rm{O}}{\rm{H}}}^{-}+{{\rm{C}}{\rm{l}}}^{-}=\equiv {{{\rm{T}}{\rm{i}}{\rm{O}}{\rm{H}}}_{2}}^{+}{\rm{\_}}{{\rm{C}}{\rm{l}}}^{-}+{{\rm{O}}{\rm{H}}}^{-}$$

Here (Figure 2(a)), …H^+^ and …OH^−^ indicate protons and hydroxyl ions in a diffuse swarm, respectively, whereas _Na^+^ and _Cl^−^ indicate Na^+^ and Cl^−^ ions that form outer-sphere complexes with the TiO_2_ NP surfaces, respectively. To maintain electroneutrality, H^+^ and OH^−^ accumulate in the diffuse swarm^26^; thus, the accumulated H^+^ and OH^−^ are postulated to be replaced gradually by Na^+^ and Cl^−^ diffusion into the TiO_2_ NP aggregates until equilibrium is attained between the complexed ions and those ions in bulk water.

**Figure 3:**
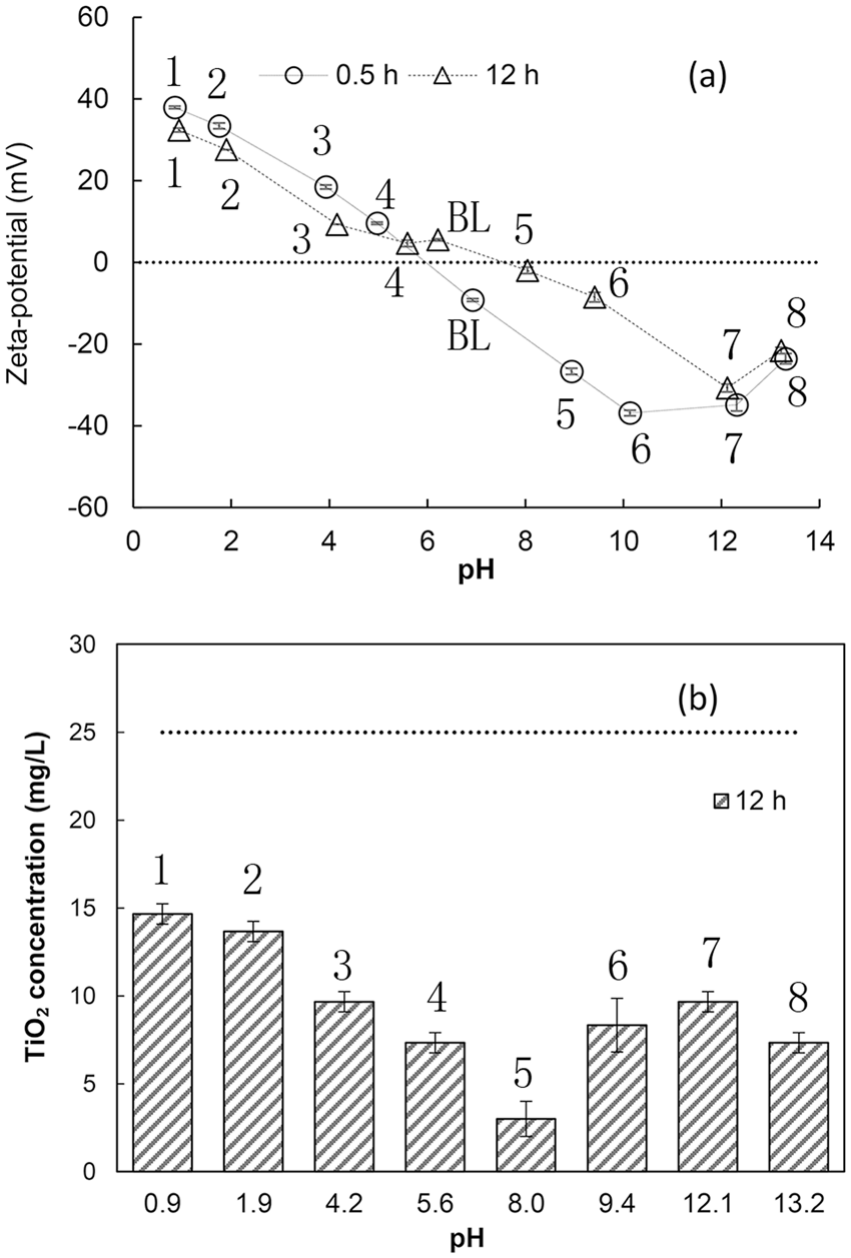
Zeta potential and pH of TiO_2_ NP suspension at 0.5 h and 12 h. (**a**) Zeta potential versus solution pH and (**b**) TiO_2_ NPs suspension at 0.5 h (25 mg/L) and at 12 h. Error bars indicate 1 standard deviation. The numbers in the figures indicate the same experiments at 0.5 h and 12 h. BL is the blank sample without pH adjustment.

Figure 3(a) shows the zeta potential at various pH values at 0.5 h and 12 h. The pH and zeta potential change after 12 h of mixing and settling. TiO_2_ NPs at 12 h are suspended in the supernatant after 12 h of mixing and 10 min of settling. The pH shifted closer to pH_pzc_, which indicates that TiO_2_ NPs exhibit a buffering capacity, albeit marginal. As mentioned previously, changes in zeta potential above pH_pzc_ were greater than those below pH_pzc_. Hence, the zeta potentials shifted closer to the zero zeta potential line. As a result, complexation of small amounts of Na^+^ or Cl^−^, e.g., at less than 10 μmol/m^2^, can affect the zeta potential significantly, and make TiO_2_ NPs prone to destabilization. Nur *et al*. investigated the aggregation of TiO_2_ nanoparticles at different concentrations of KCl and CaCl_2_ electrolytes^32^, and found that Cl^−^ anions influence the zeta potential and isoelectric point of the TiO_2_ nanoparticles. Because the absolute values of zeta potential were lowered by ion complexation, the pH_pzc_ of TiO_2_ NPs appeared to be shifted slightly to a high pH, as can be observed from the line that connects the zeta potential values at 12 h. However, the IS and Na^+^ and Cl^−^ concentrations differed between these experiments, and the analytical errors were high at zeta potential values near zero. Thus, it is more difficult to estimate pH_pzc_ after 12 h compared with the estimation at 0.5 h. Figure 3(b) shows the residual TiO_2_ NPs at 12 h. The amounts of residual TiO_2_ NPs agree fairly well with the 12-h zeta potential values; namely, the residual TiO_2_ NP concentration increased with increasing absolute zeta potential.

### Effect of Sodium and Calcium Ions

**Figure 4:**
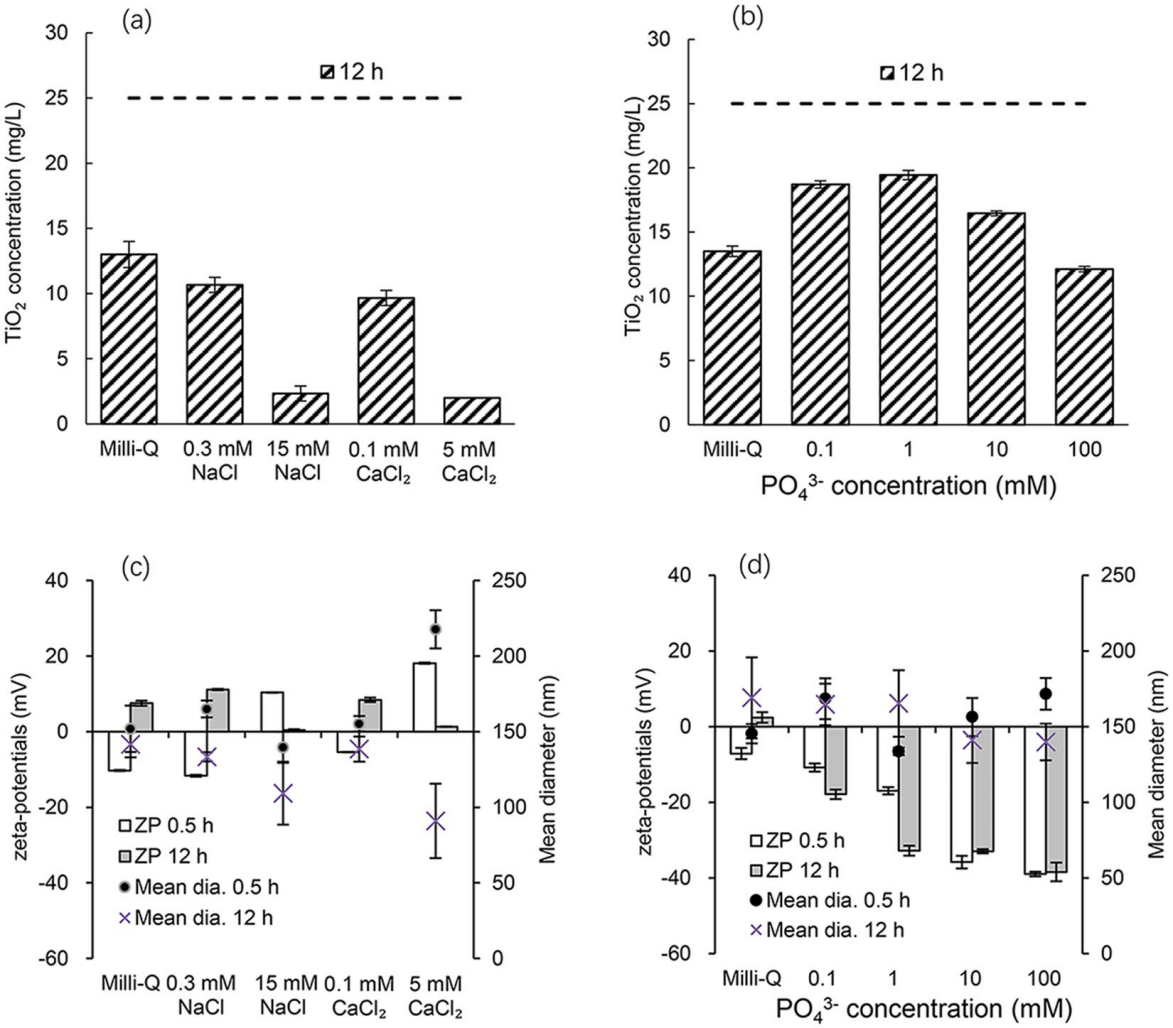
TiO_2_ NP concentration at 0.5 h and 12 h (**a**), (**b**), and zeta potential and particle diameter (**c**), (**d**). Sodium chloride or calcium chloride solutions (pH 5.4–6.3) (**a**), (**c**), and phosphate solutions (pH 8.4–9.2) (**b**), (**d**). Initial TiO_2_ NP concentration 25 mg/L. Error bars indicate 1 standard deviation.

Figure 4 shows the effect of IS on TiO_2_ NP stability. The concentration of TiO_2_ NPs in Milli-Q water at 12 h decreased from 25 mg/L to ~13 mg/L (Fig. 4(a)), whereas those in the 0.3 mM NaCl and 0.1 mM CaCl_2_ solutions (equivalent to 0.3 mM NaCl ionic strength) were 11 mg/L and 10 mg/L, respectively. At an IS of 30 mM, the TiO_2_ NP concentrations were significantly less at approximately 2 mg/L in the 15 mM NaCl and 5 mM CaCl_2_ solutions because of the TiO_2_ NPs destabilization. These results agree with data from the zeta potential measurements in Fig. 4(c). For solutions of 0.3 mM NaCl and 0.1 mM CaCl_2_, the zeta potentials shifted from slightly negative values at 0.5 h to slightly positive values at 12 h; the same observation was made in Milli-Q water. Conversely, the zeta potentials shifted from positive to nearly zero at 12 h in solutions of 15 mM NaCl and 5 mM CaCl_2_. These results indicate that at low ion concentrations, ion complexation affects the surface charge, whereas at a high ion concentration, an increase in IS results in a compaction of the electric double layer and TiO_2_ NP destabilization. At a high IS, the particle sizes increased at 0.5 h for 5 mM CaCl_2_ and then decreased at 12 h. However, for experiments conducted at low IS, i.e., at 0.3 mM NaCl and 0.1 mM CaCl_2_, the decrease in particle size was less. These results indicate that, after aggregation and settling at high IS, particles smaller than those before aggregation are retained as residual particles in the supernatant.

**Figure 5:**
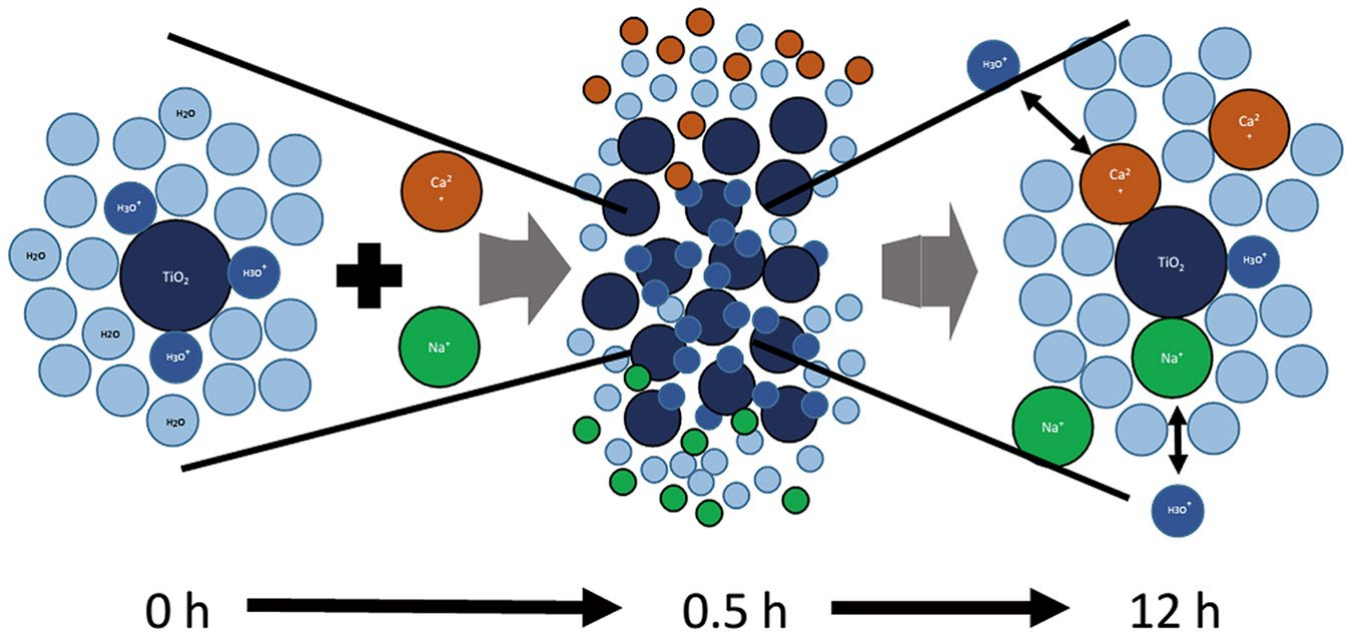
Slow process of ion complexation in 12 hours.

**Figure 6:**
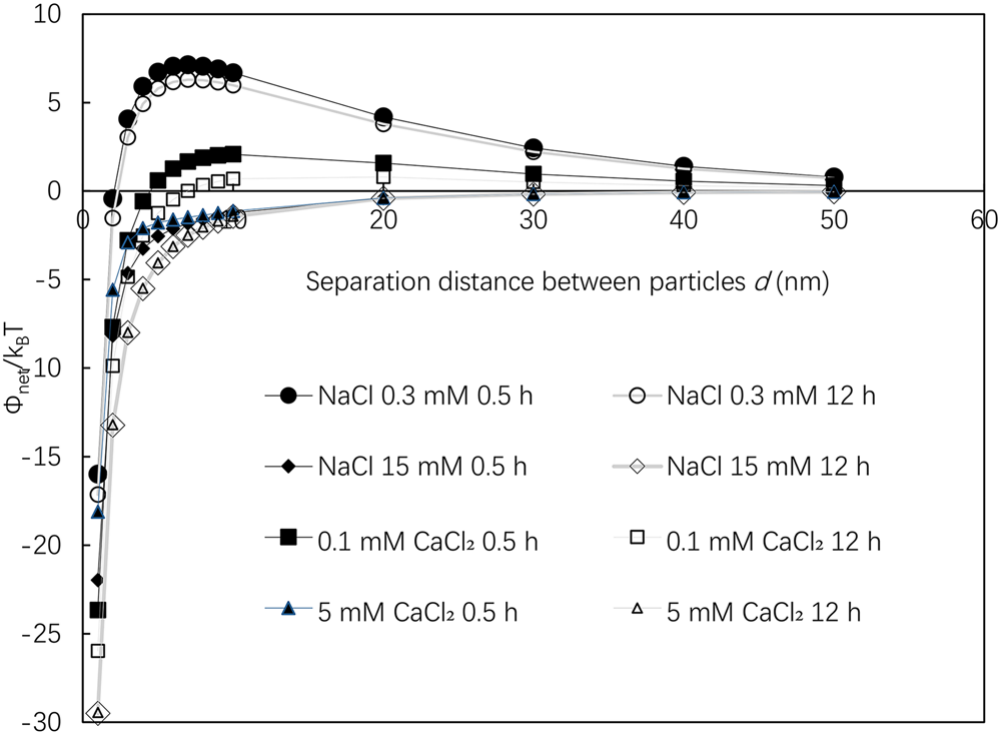
DLVO interaction energies for NaCl and CaCl_2_ solutions.

Although the application of DLVO theory to experimental results such as those in this study requires some assumptions and simplification, in general, it can describe the aggregation and deposition behavior of nanoparticles semi-quantitatively^11^. Figure 5 shows the slow process of the ion diffusion during the 12 hours. Figure 6 shows the DLVO interaction energies for NaCl and CaCl_2_ solutions. In all cases, the interaction energy decreased at 12 h compared with that at 0.5 h, and this is attributed to Na and Ca complexation. The IS was the same at 0.5 h and 12 h; thus, the changes are attributed only to ion complexation and not to changes in IS. Thus, the effects of ion complexation are evaluated independently from those of IS. At high IS values, no energy barrier was observed in solution, i.e., 15 mM NaCl and 5 mM CaCl_2_, at 0.5 and 12 h. However, at a low ionic strength, small energy barriers of approximately 7 k_B_T and 2 k_B_T were observed at 0.5 h in 0.3 mM NaCl and 0.1 mM CaCl_2_, respectively, although both energy barriers decreased after 12 h. Because these energy barriers were not sufficiently large, TiO_2_ NPs were destabilized gradually, and some settled after 12 h of mixing. At the same IS, CaCl_2_ was more effective than NaCl at lowering the energy barrier, although a difference in particle settling was not observed in Fig. 4. The surface charge of TiO_2_ NPs became positive because of Na^+^ or Ca^2+^ complexation; and thus, dominant ions in the electric double layer are Cl^−^, the concentration of which in a 0.3 mM NaCl solution was slightly higher than that in a 0.1 mM CaCl_2_ solution. Hence, the destabilization effect of Ca^2+^ was nearly the same as that of Na^+^ at the same ionic strength, which differs from the reported effects of Ca and Na ions by French *et al*.^19^.

### Effect of Phosphate Ions

At a low concentration (0.1–1 mM), PO_4_^3−^ stabilized TiO_2_ NPs; however, the stabilization effect decreased at a high PO_4_^3−^ concentration (10 mM and 100 mM), as shown by the increased TiO_2_ NP concentration after 12 h of mixing and settling (Fig. 4 (b). The maximum stabilization of PO_4_^3−^ is a result of the combined effects of PO_4_^3−^ complexation and increased IS. Because of the limited complexation sites on the TiO_2_ NP surface, no further increase in negative charge was observed at PO_4_^3−^ concentrations above 10 mM, whereas the IS increased with increasing PO_4_^3−^. Thus, the effect of high IS to compress the electric double layer overwhelmed the effect of particle repulsion, which is attributed to a negative surface charge.

The zeta potential decreased from 0.5 h to 12 h at low PO_4_^3−^ concentrations of 0.1–1 mM, whereas the zeta potential did not decrease significantly between 0.5 h and 12 h at 10–100 mM. This result suggests that ion complexation and equilibration between complexed ions and ions in the bulk solution are dependent on concentration, and that the reaction is diffusion-limited. The zeta potentials of TiO_2_ NPs became more negative with an increase in phosphate concentration from approximately −18 mV at 0.1 mM to approximately −40 mV at 100 mM, but the zeta potential differed only slightly in solution at 10 mM and 100 mM.

The energy barrier of the DLVO model was slightly high at ~9 k_B_T at 0.1 mM PO_4_^3−^ and then increased to a maximum energy barrier of ~28 k_B_T at 1 mM. With further increase in PO_4_^3−^ concentration, it decreased gradually to 5–8 k_B_T at 10 mM, and no energy barrier was observed at 100 mM (Figure S4). An energy barrier of ~15 k_B_T is considered to be sufficiently high to maintain particle dispersion^21^. These results from the DLVO interaction energy calculation agreed with those obtained from aggregation and settling, as shown in Fig. 4(b).

### Destabilization in Lake Water

**Figure 7:**
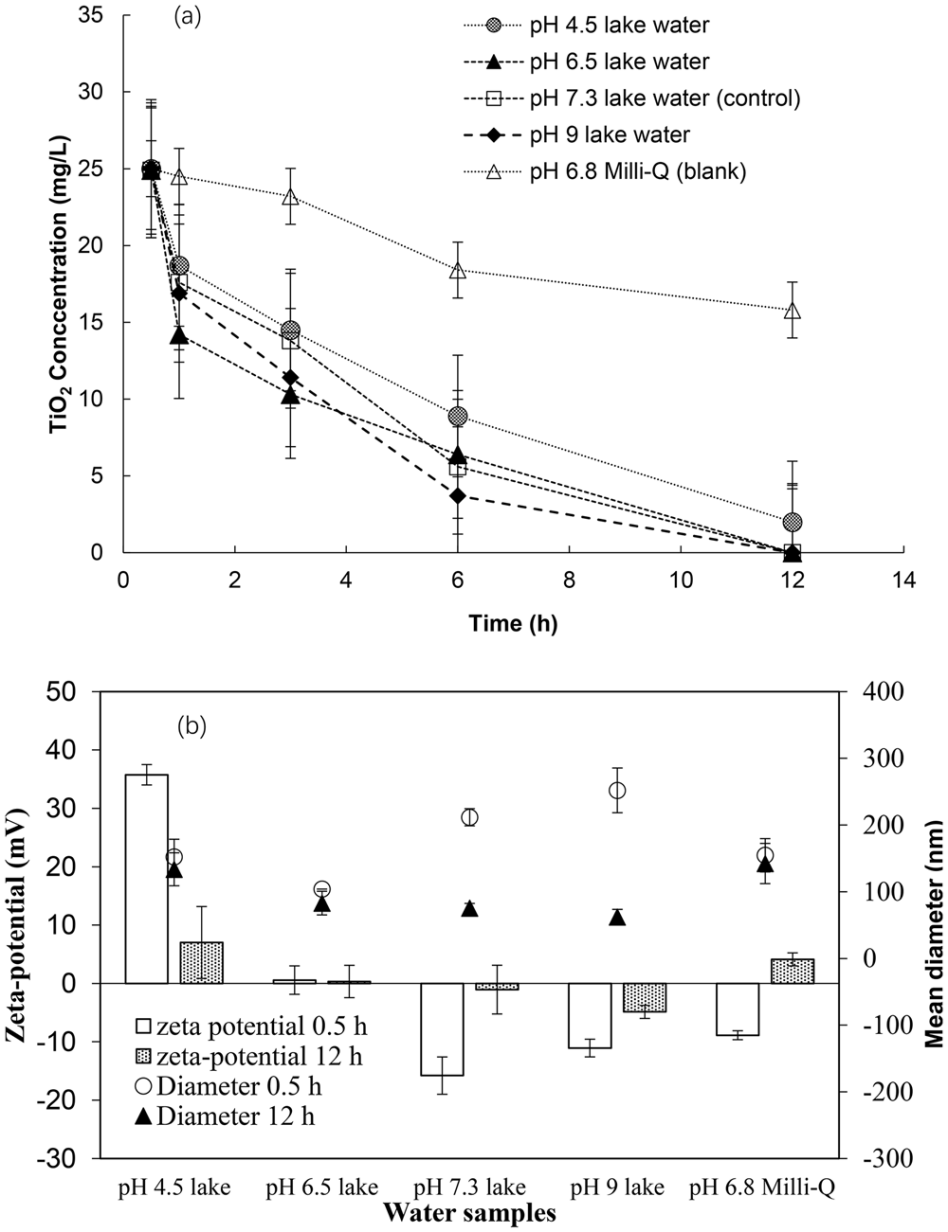
(**a**) Change in TiO_2_ concentrations of suspensions in lake water (**b**) zeta potentials at 0.5 h and 12 h of equilibration and mean diameters measured at 0.5 h. Initial TiO_2_ concentration was 25 mg/L. Error bar indicates 1 standard deviation.

Figure 7(a) shows the change in suspended TiO_2_ NPs in the lake water with or without buffer, and the results from the blank experiment in Milli-Q water. In lake water, more than 90% of the TiO_2_ NPs settled after 12 h under all experimental conditions, whereas ~60% (15.8 mg/L) of the TiO_2_ NPs remained in the supernatant in Milli-Q water. Despite the addition of 20 mM PO_4_^3−^, the rate of aggregation and settling was most rapid at pH 6.5 because the pH was closest to the pH_pzc_ from the beginning (0.5 h) of the experiment, as shown in Fig. 3(a). Although the complexation of PO_4_^3−^ in Milli-Q water caused a lowering in zeta potential of TiO_2_ NPs in Milli-Q water as shown in Fig. 4(d), there was no effect of PO_4_^3−^ addition on the change in zeta potential of TiO_2_ NPs in the lake water. This result is attributed to the fact that the complexation of PO_4_^3−^ with the limited numbers of sites on the TiO_2_ NPs was interrupted by competition among various anions and organic matter, and because the negative surface charge of the complexed PO_4_^3−^ was shielded by the complexation of other ions and a compaction of the electrical double layer, which is caused partly by an increased ionic strength from PO_4_^3−^ addition. In the lake water, the rates of aggregation and settling of TiO_2_ NPs were of the order of pH 6.5 > pH 9.0 > pH 7.3 > pH 4.5. A paired sample t-test showed that the differences within the first 6 h were significant (P < 0.05). This order agrees with the zeta potentials of these solutions; namely, TiO_2_ NPs in lake waters have smaller zeta potentials and aggregate and settle more rapidly compared with other NPs. The slow aggregation and settling of TiO_2_ NPs in the lake waters were attributed to relatively high zeta potentials at 0.5 h, which approached zero after 12 h of gentle mixing. In the control experiment at pH 7.3, without the addition of any buffer solution, a strong negative charge of −15 mV was possibly neutralized by the complexation of ions in the lake water. Thus, the change in zeta potential during mixing for 12 h and slow aggregation observed in the synthetic waters was also observed in the lake waters. These results contrast with the rapid aggregation in a jar test with a coagulant dose under strong mixing conditions^9^. Based on the results obtained for synthetic waters, slow aggregation in this study was postulated to be caused by diffusion-limited complexation because of the formation of TiO_2_ NP aggregates and gentle mixing. The results obtained herein are more relevant for the prediction of the aggregation of commercially used NPs in natural water bodies compared with those obtained under strong mixing conditions as reported previously^17, 19, 22, 27^.

Figure 7(b) shows the particle sizes of the TiO_2_ NPs in the supernatant. The particle size at 0.5 h was smallest in the pH 6.5 solution. These results are indicative of the rapid aggregation and settling of large aggregates at pH 6.5. After 12 h, the particle sizes became smaller than those at 0.5 h in all solutions. At pH 7.3 and pH 9, the residual particle sizes were smaller than those at pH 6.5.

Figure S5 shows the results of the DLVO interaction energies of TiO_2_ NPs in the lake water. As mentioned previously, despite the addition of a 20 mM PO_4_ buffer solution, the zeta potential of TiO_2_ NPs was nearly zero at pH 6.5; hence, no energy barrier was observed in the DLVO model. Moreover, even at pH 9.0, the energy barrier was also negligible. For lake water without any buffer at pH 7.3, a small energy barrier was observed at 0.5 h, but it decreased after 12 h. A very high energy barrier of 45 k_B_T in the pH 4.3 solution at 0.5 h decreased to an almost negligible value at 12 h; thus, most TiO_2_ NPs settle after 12 h, even at pH 4.5.

## Conclusion

An understanding of TiO_2_ NP behavior in natural water sources is important for human health. The presence of various salts, such as Na^+^, Cl^−^, and PO_4_^3−^ could be expected in natural waters and may affect TiO_2_ NP stability. This study has progressed the understanding of TiO_2_ NPs in the presence of certain ions, and under conditions that simulate mixing in certain natural water conditions. It has also highlighted the potential dangers that could result should small-particle aggregates remain suspended after TiO_2_ NP settling. The behavior and hazards of such particles should be investigated further. The following specific areas should be considered.

The accumulation of inert ions (Na^+^ and Cl^−^) in the diffuse layer which was not discussed in other studies was supposed to be the main reason that aggregation occurred slowly and continuously. An understanding of the slow complexation and aggregation as they may be attributed to the diffusion of ions (Na^+^ and Cl^−^) into the aggregates, followed by equilibration with bulk water, provides important information towards being able to predict subsequent destabilization, removal of large aggregates, and possibly the amount of residual TiO_2_ NP aggregates of a small particle size. Future work should include studies on the residual particles after destabilization and settling.

The knowledge that PO_4_^3−^ stabilizes TiO_2_ NPs at low concentrations of less than or equal to 10 mM, but destabilizes NPs at 100 mM is useful for understanding the TiO_2_ NP behavior in environments in which PO_4_^3−^ is present. An addition of 20 mM PO_4_^3−^ into lake water led to TiO_2_ NP destabilization because of the negligible effect of PO_4_^3−^ adsorption on the surface charge, which is overwhelmed by the effects of a high ionic strength. These results indicate that DOM and PO_4_^3−^ affect the stabilization of TiO_2_ NPs in natural waters marginally, and can be quantitively explained by the visualized DLVO-theory-based energy interaction figures. An understanding of the TiO_2_ NP behavior with PO_4_^3−^ addition may allow for controlled TiO_2_ NP removal. Additional future work may include understanding whether the addition of PO_4_^3−^ to TiO_2_ NP suspensions could be used advantageously in the removal of aggregates of small particle size.

### Data Availability Statement

All data supporting this study are provided as supplementary information accompanying this paper.

## Electronic supplementary material

Supplementary Information
