# Supplementary material for: Properties of residual titanium dioxide nanoparticles after extended periods of mixing and settling in synthetic and natural waters
Source: Sci Rep. 2017 Aug 30;7:9943. doi: 10.1038/s41598-017-09699-9 (PMC5577155; doi:10.1038/s41598-017-09699-9)
Supplement: Supplementary file 1 — Supplementary Information [file 41598_2017_9699_MOESM1_ESM.pdf]

Supplementary Information

Properties of residual titanium dioxide nanoparticles after extended periods of mixing and settling in synthetic and natural waters

Chunpeng Zhang[1,3], Jenyuk Lohwacharin[2,3]， Satoshi Takizawa[3]

*Corresponding Author:

[1] Jilin University, Key Laboratory of Groundwater Resources and Environment, Ministry of Education, No.2519, Jiefang Road, Changchun 130021, China.

[2] Chulalongkorn University, Department of Environmental Engineering, Faculty of Engineering, Phayathai Rd., Wangmai Pratumwan, Bangkok 10330, Thailand.

[3] the University of Tokyo, Department of Urban Engineering, Graduate School of Engineering, 7-3-1 Hongo, Bunkyo-ku, Tokyo 113-8656, Japan.

Correspondence and requests for materials should be addressed to CP.Z (email: zhang_cp@jlu.edu.cn) or S.T. (email: takizawa@env.t.u-tokyo.ac.jp)

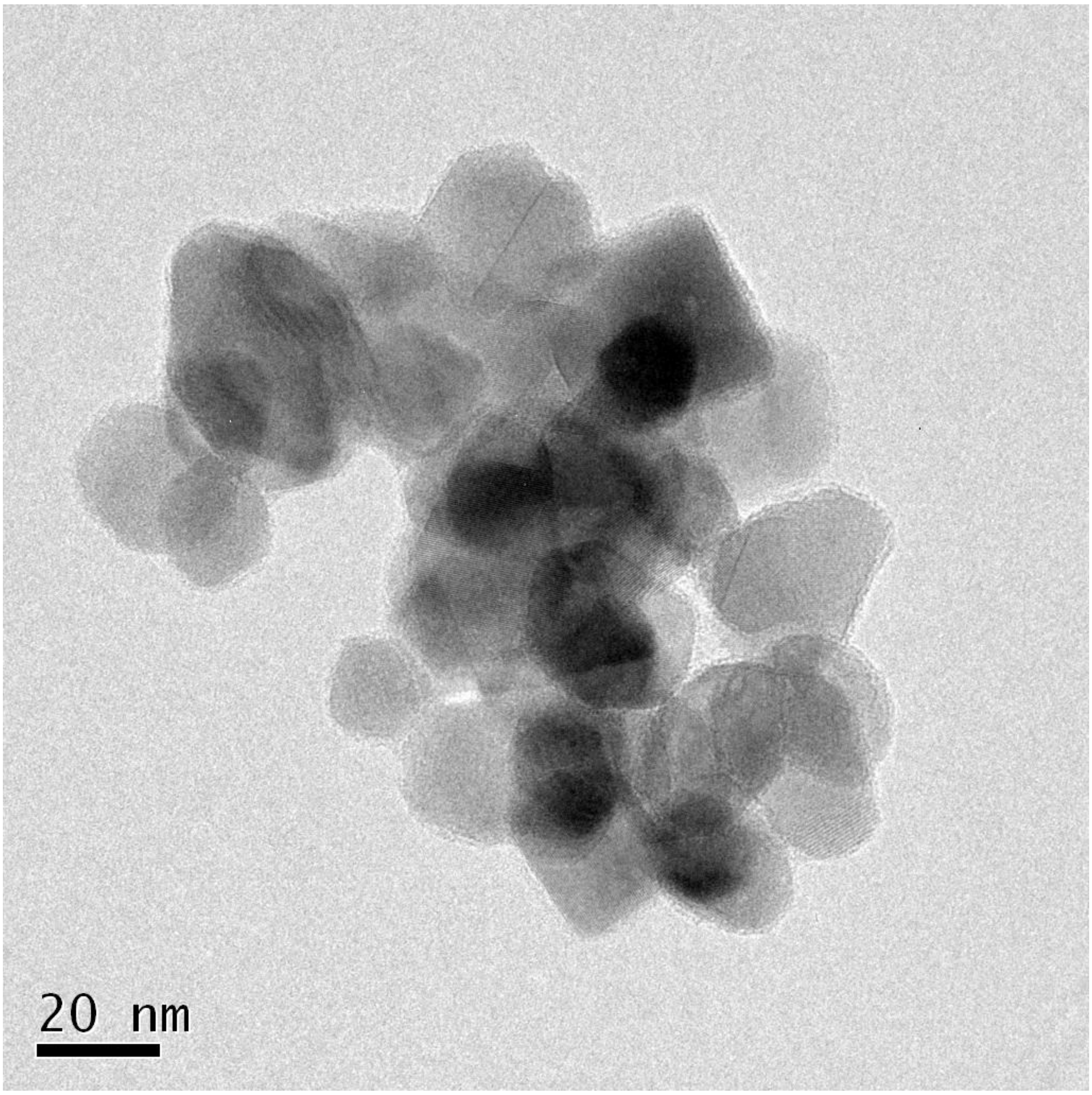

Figure S1. Transmission electron micrograph of TiO_2 NPs.

Primary particle size of ~25 nm as reported by the manufacture. The aggregate size was ~100 nm. Photo taken using a JEOL 2000EX2 transmission electron microscope at 200 kV.

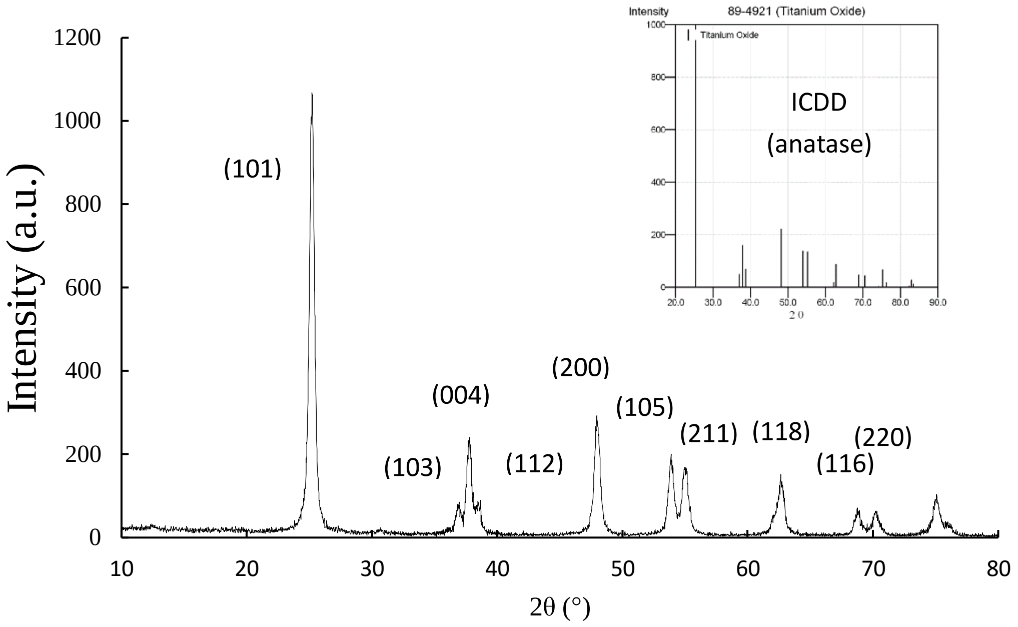

Figure S2. X-ray powder diffraction spectrum for TiO_2 NPs.

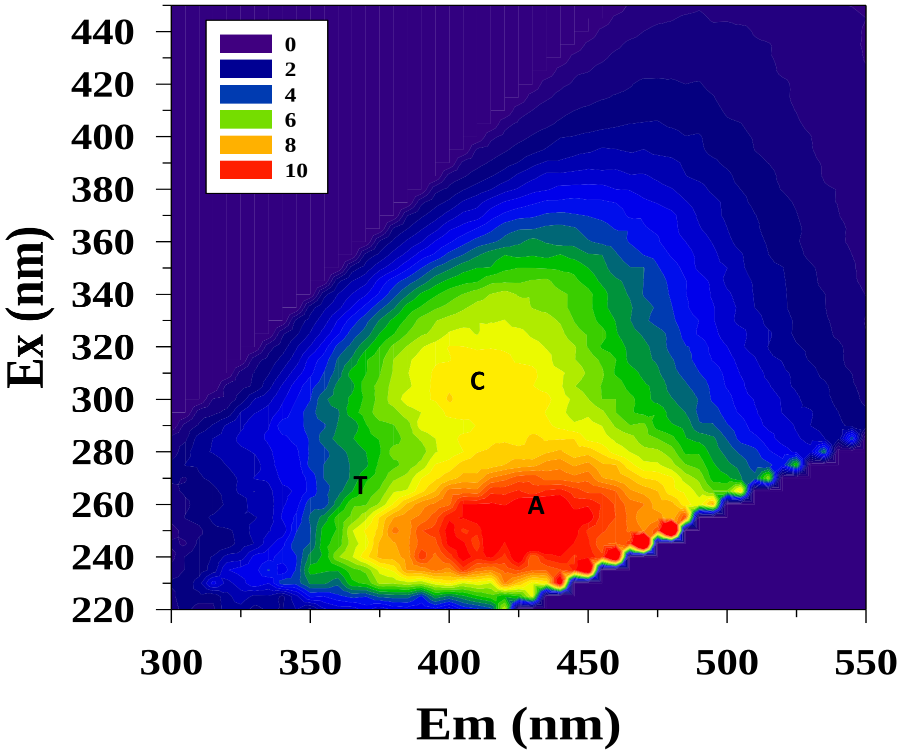

Figure S3. EEM spectra of Lake Kasumigaura water.
Intensity in Raman unit. C represents Humic-like OM, A represents Fulvic-like OM, T represent S Tryptophan-like OM.

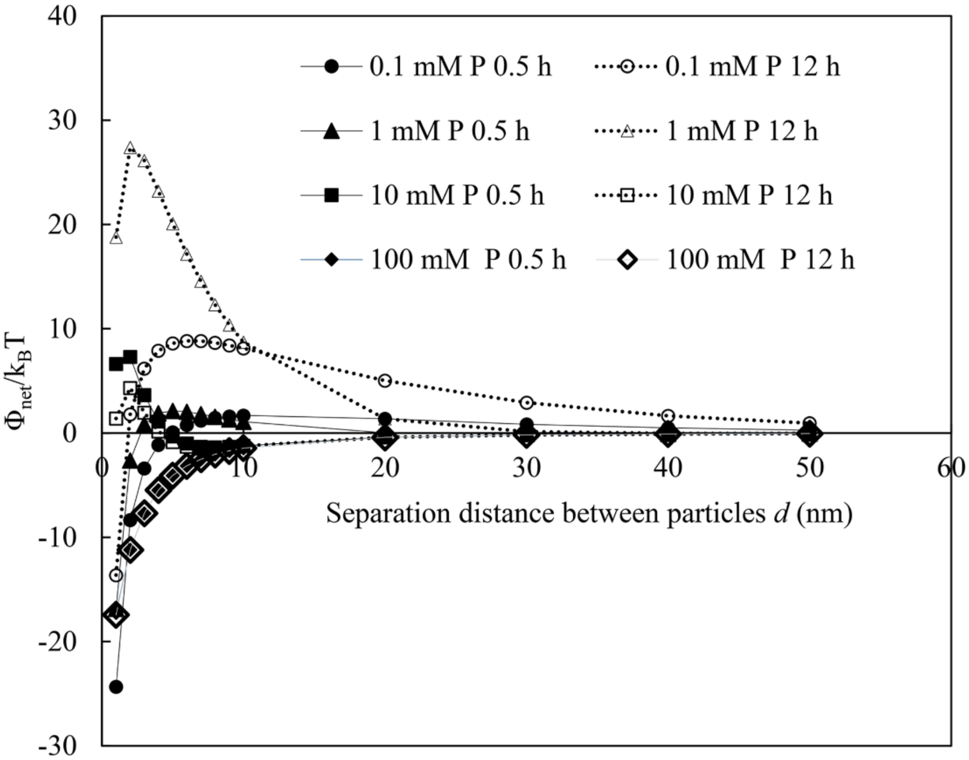

Figure S4. DLVO interaction energies for PO_4^{3-} solutions.

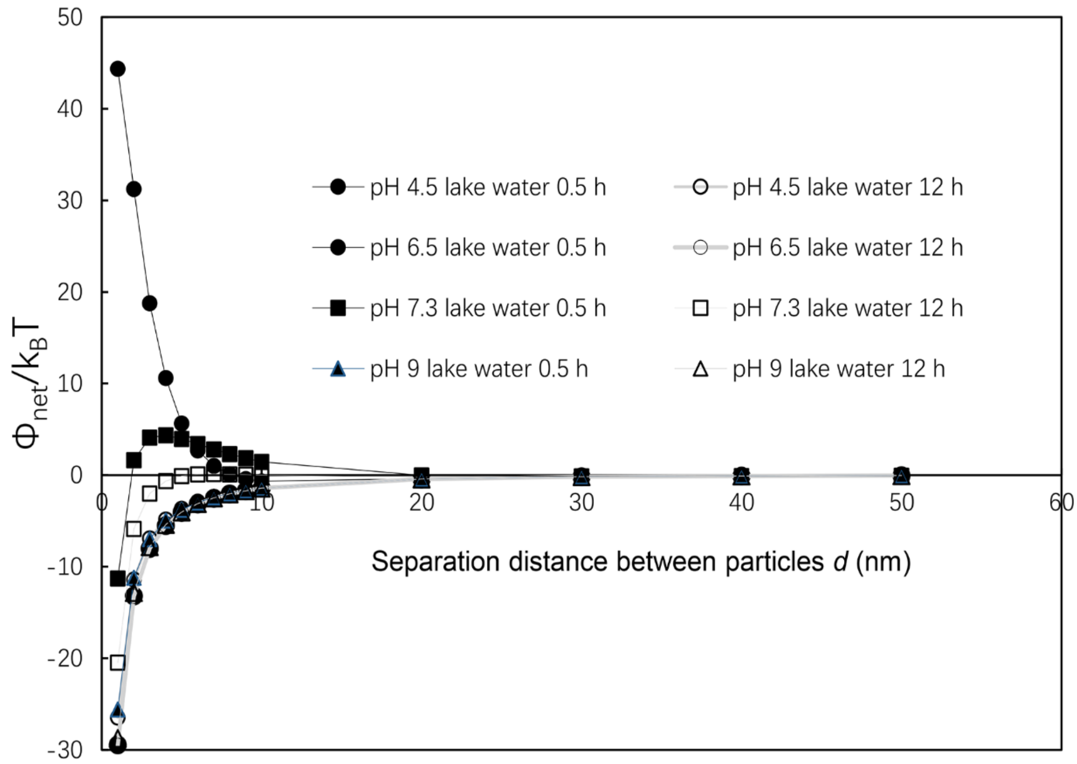

Figure S5. DLVO interaction energies of TiO_2 NPs in lake water.

Table S1 Kasumigaura lake water parameters

Parameters	Data
pH	8.50
Conductivity (µS/cm)	275.3
Alkalinity (mg/L as $CaCO_3$)	54
Turbidity (NTU)	20.3
DOC (mg/L)	3.50±0.04
UV_{254} (1/cm)	0.066±0.002
$SUVA_{254}$ (L/mg.m)	1.89
Total nitrogen (mg/L as N)	0.94
Anion concentrations	
Chloride (mg/L)	30.92
Bromide (mg/L)	0.123
Nitrate (mg/L)	8.07
Sulfate (mg/L)	22.75
Cation concentrations	
Magnesium (mg/L)	6
Potassium (mg/L)	5
Calcium (mg/L)	11
Maganese (mg/L)	ND
Iron (mg/L)	ND
Sodium (mg/L)	20
Aluminium (mg/L)	0.2
Note: ND = Not detectable	

Table S2. Hamaker constant (A) of anatase TiO_2 in different media

No.	Hamaker Constant A (10^{-20}J)	Reference	Note
1	3.7	Gómez-Merino *et al*., 2007	
2	3.5	Gómez-Merino *et al*., 2007	estimated through contact angle
3	2.5	Clark *et al*., 2009	
4	3.7	Guo *et al*., 1985	
mean	3.4		

References:

Gómez-Merino, A.I., Rubio-Hernández, F.J., Velázquez-Navarro, J.F., Galindo-Rosales, F.J., Fortes-Quesada, P, 2007. The Hamaker constant of anatase aqueous suspensions. Journal of colloid and interface science, 316, 451-456.

Clark. M. M., 2009. Transport Modeling for Environmental Engineers and Scientists, Wiley, p. 104.

Guo, J., Tiu, C., Uhlherr, P. H. T., Fang, T.-N., 2003. Yielding behaviour of organically treated anatase TiO_2 suspension, Korea-Australia Rheology Journal, 15(1), 9-17.
